# A Rare Presentation of Undiagnosed Systemic Syphilis: A Case Report and Review of Literature

**DOI:** 10.7759/cureus.27911

**Published:** 2022-08-12

**Authors:** Ariel Ruiz de Villa, Asad A Haider, Leora Frimer, Amina Lleshi, Yvette Bazikian

**Affiliations:** 1 Internal Medicine, Hospital Corporation of America (HCA) & University of Central Florida (UCF) College of Medicine, North Florida Regional Medical Center, Gainesville, USA

**Keywords:** syphilis rash, syphilis uveitis, neurosyphilis, secondary syphilis, syphilis

## Abstract

Within the specialties of infectious diseases and dermatology, few rashes involve the palms and soles. The syphilitic rash has a pathognomonic association with these body surfaces and signals physicians to investigate this disease. However, the distinct presentations and symptoms associated with syphilis and the various stages of the disease make it diagnostically challenging. We herein report a rather intricate and unusual case of a patient who presented with a new-onset headache and blurred vision and a two-month history of diffuse pruritic maculopapular rash sparing the palms and soles. Several physicians had not established a diagnosis in the outpatient setting. Inpatient workup eventually revealed that the patient was suffering from secondary syphilis with neurological and ocular involvement. Management included a prolonged course of intravenous penicillin G leading to a complete recovery. We share images of the skin findings and the details of the intricate workup and hospital course, as well as a review of the literature.

## Introduction

Syphilis, caused by the spirochete *Treponema pallidum*, is primarily transmitted by sexual contact and can also be transmitted across the placenta during childbearing [1]. With the discovery of penicillin and the overall progress of research and medicine, syphilis now affects fewer people than before [1]. Syphilis is known as the “great imitator.” It rears its head in an array of manifestations at different stages and can re-occur during a patient’s life, making it diagnostically challenging [2]. The hallmarks of secondary syphilis are a flulike illness and a diffuse, often symmetrically distributed maculopapular rash that typically involves the palms and soles [2].

Herein, we report a rare presentation of secondary syphilis, with a rash sparing the palms and soles, lasting for over two months. After consulting an outpatient dermatologist and a primary care physician, the patient was told she had a viral exanthem, but a diagnosis was not established. The patient resorted to coming to the hospital after experiencing severe headaches and vision impairment. During her hospitalization, a comprehensive workup including skin biopsies, serum, and cerebrospinal fluid analysis revealed evidence of syphilis infection. This case report aims to portray the varied ways in which syphilis can be present and the importance of its inclusion in the differential of dermatologic abnormalities. A review of the literature and a comprehensive discussion on the topic is provided.

## Case presentation

A 46-year-old female with a past medical history of migraine headaches and antiphospholipid antibody syndrome with remote deep venous thrombosis and miscarriages, presented with a one-day history of severe frontal headache and left-eye blurred vision and pain. Additionally, she noted a two-month history of diffuse, pruritic, maculopapular rash that started on the abdomen and extended to the entire body, sparing the palms and soles. She also reported worsening fatigue and nonspecific arthralgias. The patient attributed her symptoms to a migraine headache. However, she noted that over-the-counter migraine medications and rest did not result in improvement of her symptoms. She noted pruritus, which was only mildly relived by diphenhydramine and loratadine. She was evaluated by outpatient dermatology and told that her rash was a viral exanthem that would resolve spontaneously. Two weeks prior to the presentation, she had a complete viral panel done outside of our facility that resulted only for cytomegalovirus (CMV) antibodies.

Otherwise, the patient did not endorse any chest pain, abdominal pain, genitourinary or digestive issues, or syncope. She denied sick contacts, a history of sores, recent insect bites, or exposure to animals. She reported that her husband was asymptomatic. Additionally, she reported a monogamous relationship and no other sexual partners. The patient smoked tobacco daily and reported occasional marijuana use. She denied the use of prescription medication or illicit drug use.

**Figure 1:**
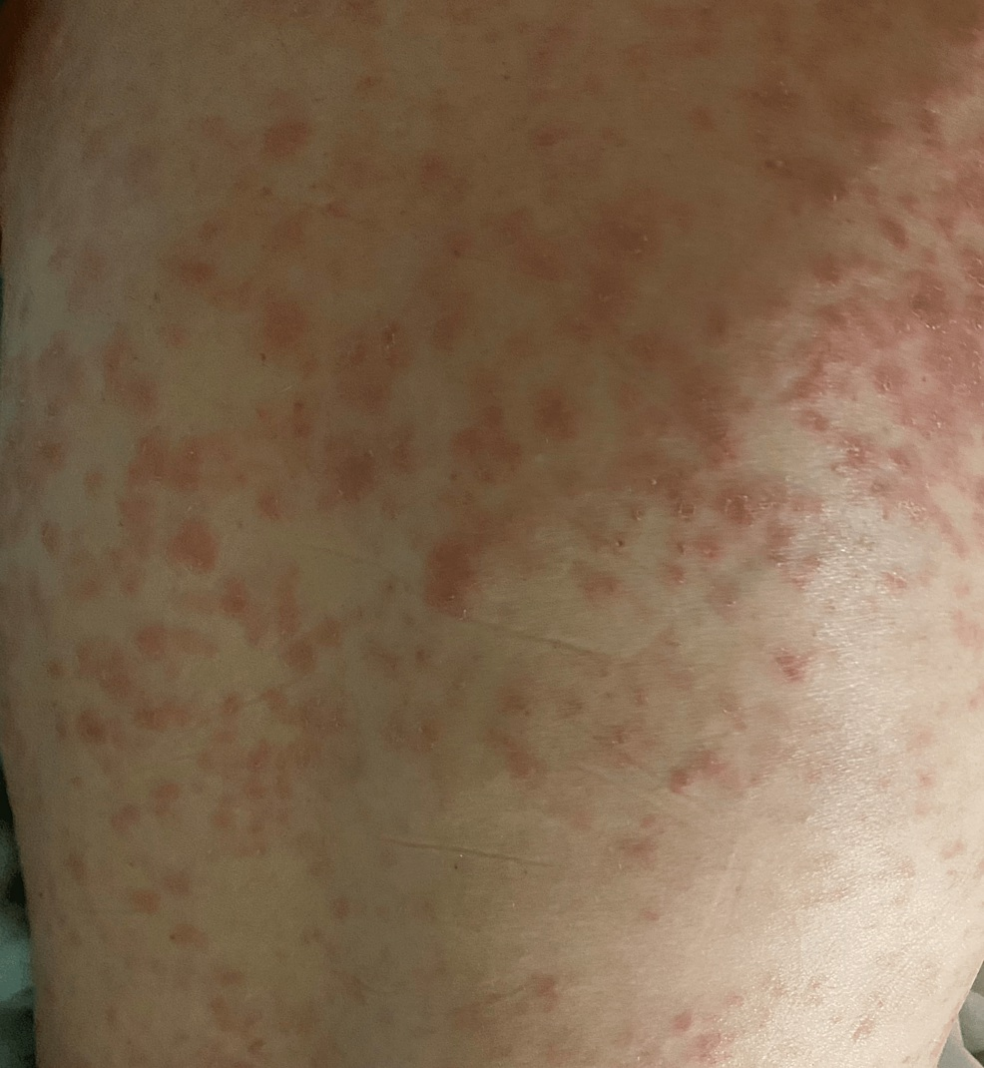
Patient's physical exam findings of the back showing a diffused, non-confluent and erythematous maculopapular rash.

**Figure 2:**
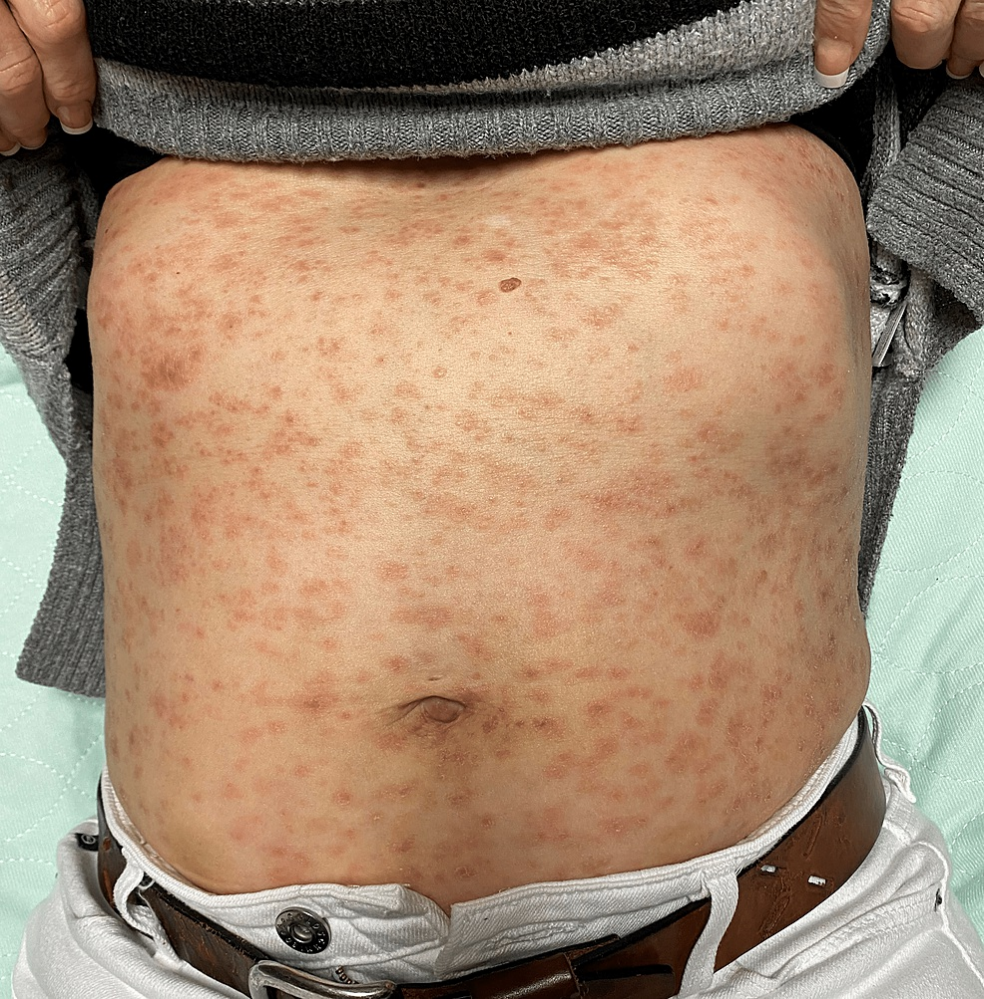
Patient's physical exam findings of the abdomen showing a diffused, non-confluent and erythematous maculopapular rash.

Physical examination was consistent with a diffuse maculopapular rash involving more than 90% of her skin, only sparing the palms, soles, and gluteal folds. Figure 1 and Figure 2 are images of the patient’s rash, back, and abdomen, taken on admission. Both conjunctivae appeared mildly injected, with increased lacrimation of the left eye. Pupils were regular with 3 mm in diameter bilaterally, with minimal constriction under bright light exposure. They appeared to accommodate nearby objects appropriately. The patient’s gait appeared unsteady. Otherwise, the remainder of the physical examination, including vital signs, was unremarkable and within normal limits. Admission labs are presented in Table 1. An abdominal ultrasound was normal. The CT abdomen showed mild nonspecific hepatosplenomegaly.

**Table 1:** Pertinent laboratory data at the time of presentation. AST: aspartate aminotransferase; ALT; alanine aminotransferase; BUN; blood urea nitrogen; MCV: mean corpuscular volume.

Tests	Results	References	Units
White blood count	6.7	4.0-10.5	10³ uL
Hemoglobin	8.6	11.2-17.5	g/dL
Hematocrit	34.1	40.1-51	%
Platelets	658	150-400	10³ uL
MCV	63.9	79.0-92.2	fl
Sodium	137	136-145	mmol/L
Potassium	3.9	3.5-5.1	mmol/L
Chloride	106	98-107	mmol/L
Carbon dioxide	26	21-32	meq/L
Glucose	84	74-106	mg/dL
BUN	23	7-18	mg/dL
Creatinine	0.86	0.6-1.30	mg/dL
Albumin	2.6	3.4-5.0	g/dL
Total bilirubin	0.2	0.2-1.0	mg/dL
AST	95	15-37	units/L
ALT	89	13-56	units/L
Alkaline phosphatase	333	45-117	units/L
Lactic acid	0.58	0.4-2.0	mmol/L
C-reactive protein	5.4	0.8-1.0	mg/dL

Given the presentation of rash, hepatosplenomegaly, thrombocytosis, anemia, and headache, the admitting physician diagnosed the patient with the maculopapular rash of unknown etiology as well as atypical migraine. The patient was admitted and treated with IV ganciclovir for possible CMV infection and infectious disease was consulted. On further evaluation, the differential was broadened to include viral infection, autoimmune disease, vasculitis, and zoonotic infection. Several serological labs were obtained and presented in Table 2. The patient was empirically treated with doxycycline and ivermectin to broadly cover for rickettsia and parasitic infections. A biopsy of the rash was taken and sent for pathological evaluation.

**Table 2:** Serologic labs. Antibodies may include IgM/IgG as appropriate with context. PCR: polymerase chain reaction; CMV: cytomegalovirus; RPR: rapid plasma regain.

Lab	Result
Syphilis serum PCR	Positive
RPR	Positive
RPR titer	1:32
Treponema palladium antibody	Positive
Chlamydia urinary test	Negative
Cryptococcal antigen	Negative
CMV IgG antibodies	Positive
CMV DNA	Negative
HIV	Negative
Hepatitis A antigen	Negative
Hepatitis B surface antigen	Negative
Hepatitis C antibody	Negative
Rickettsia antibody	Negative
Herpes simplex 1 and 2 antibodies	Negative

Over the next two days, the patient appeared to worsen, with unrelenting headaches and new left-eye blindness. The patient had marked difficulty ambulating. A lumbar tap was obtained with abnormalities suggesting cerebrospinal fluid infection. The results are depicted in Table 3.

**Table 3:** Cerebrospinal fluid laboratory results. CSF: cerebrospinal fluid; VDRL: venereal disease research laboratory test.

Tests	Results	References	Units
CSF appearance	Clear/colorless	Clear/colorless	n/a
CSF white blood cell	16	0-5 cells	×10^6^/dL
CSF red blood cells	1	0-10 cells	×10^6^/dL
CSF segmented neutrophils	4	0-6	%
CSF lymphocytes	69	50-60	%
CSF total protein	71	15-45	mg/dL
CSF glucose	48	50-80	mg/dL
CSF VDRL	Negative	Negative	n/a

Labs eventually yielded positive Treponema palladium antibodies, syphilis serology, and rapid plasma regain (RPR) with titers of 1:32. Given the interpretation of CSF analysis with >5 white blood cells and elevated protein, a diagnosis of secondary syphilis and neurosyphilis was made. The patient was immediately started on continuous intravenous penicillin G. An ophthalmologist evaluated the patient under a slit lamp, and a diagnosis of bilateral syphilitic uveitis was established. A detailed ophthalmic evaluation was not available to us, only the diagnosis was provided. The patient was subsequently started on ophthalmological steroids. The skin biopsy was consistent with a hypersensitive response to an antigen, with drug or infection as a possible etiology. After two days of treatment, the patient showed signs of gradual improvement. Her headache nearly resolved, and so did the rash and the pruritus. The patients’ gait improved as well. Her visual deficits resolved within five days of treatment. A peripheral intravenous catheter was inserted for the continuation of antibiotic treatment at home for a total of 14 days. She was educated on the pathophysiology and infectious nature of her condition and what to expect in the future. It was recommended for her partner to be tested for the disease. She expressed relief that her bothersome rash had successfully been diagnosed and treated. Twenty-one days later, during a follow-up visit, the patient was back to her baseline and the rash had resolved completely.

## Discussion

Syphilis is known as “the great imitator” due to its diversity of clinical and dermatological presentations. The incidence of syphilis in the United States has risen in recent decades, with approximately 150,000 cases diagnosed every year [3]. As the incidence of syphilis infections increases, it is important for physicians to become familiar with the varying clinical presentations of the disease, especially those that deviate from classical presentations.

Syphilis is a disease that has been known for thousands of years [4]. Local inflammatory responses elicited by spirochetes are thought to be the root cause of all the symptoms of syphilis [5]. Perivascular infiltration by lymphocytes, plasma cells, and histiocytes causes endothelial cell swelling, and the secretion of cytokines, such as tumor necrosis factor (TNF) and interleukin-6 (IL-6), causes tissue damage [6]. Spirochetes are rarely seen on biopsy of skin lesions of patients with syphilis, but polymerase chain reaction (PCR) can demonstrate the presence of *Treponema pallidum*, which suggests that the rash is a direct reaction to an infecting organism [7]. Indeed, our patient did not have evidence of spirochetes on biopsy. PCR testing was not done on the samples of this patient, but the diagnosis was confirmed by serology.

Syphilis has traditionally been thought to be present in different stages: primary, secondary, latent, and tertiary [8]. However, the stages are now known to overlap and do not always present in discrete phases. In primary syphilis, a chancre develops at the site of treponemal inoculation two to six weeks after contact with an infected person. The chancre typically presents as a painless, nonpurulent ulcer with a clean base and lasts three to six weeks without treatment [8]. The chancre usually occurs in the genital areas but can occur in extragenital areas as well, such as the hands or mouth [9]. Secondary syphilis usually occurs 4-10 weeks after initial infection but can occur at any time during the disease process [8]. It typically consists of a rash of varying severity in different areas of the body, such as the palms, soles, flanks, and arms [8]. Patients may also experience sore throat, lymphadenopathy, or gray mucous lesions, known as condyloma lata usually appears around the mouth and genital areas while the mucus patches are different cutaneous manifestations of syphilis on the mucous membranes [8].

Latent syphilis is a stage in which patients are seroreactive but asymptomatic; it can occur at any time and is not always present in the disease process. Tertiary syphilis can be present in three main forms: gummatous syphilis, cardiovascular syphilis, or CNS gumma, along with tabes dorsalis and general paresis [8]. In gummatous syphilis, granulomatous-like lesions deposit on various organs, such as bone, skin, and the liver [8]. Cardiovascular syphilis usually presents as aortitis; it is the rarest of the three forms [8]. Neurosyphilis can occur at any time during the disease process, and manifestations include meningitis, focal neurological deficits, or ocular abnormalities [8]. 

The classical presentation of secondary syphilis is a rash involving the palms and soles. However, many atypical dermatological manifestations have been described in the literature (Table 4) [4,10]. Our patient’s case, to the best of our knowledge, is among the first reported cases to date of a patient with syphilis who has a diffuse maculopapular rash that spares the palms and soles. Living up to its reputation as “the great imitator,” our patient was initially thought to have a rickettsial or parasitic infection and was treated empirically with doxycycline and ivermectin. However, further serologic testing confirmed that the patient’s symptoms were due to syphilis, and she was switched to penicillin-based therapy, after which her symptoms improved quickly. This dermatological presentation might be a diffuse variant of follicular syphilis, in which syphilis lesions occur preferentially around the hair follicles, which are not present on the palms and soles [10]. It is ultimately difficult to know the definitive reason why the rash in this patient spared the palms and soles. This case demonstrates why it is important to keep syphilis in the differential diagnosis when encountering atypical dermatologic presentations.

**Table 4:** Characteristics of atypical cutaneous manifestations of syphilis.

Type of lesion	Characteristic appearance	Distribution on body
Lichenoid	Flat lesions resembling lichen planus	Back, arms, upper trunk
Vesicular	Raised, reddish base; pointed, very small	Any part of the body
Corymbiform	Large raised lesions surrounded by smaller flash lesions	Any part of the body
Follicular	Pointed papules around hair follicles	Face, back, arms, upper trunk, thighs
Psoriasiform	Scaly, colored lesions resembling psoriasis	Face, palms, soles, elbows, knees
Condyloma lata	Papules that have become macerated and covered with mucoid exudate	Groin, scrotum, volva, rectum
Rupial	Large pustules with crusting	Any part of the body
Annular	Papules with a circinate configuration and surrounding rings	Any part of the body
Frambesiform	Hypertrophic, papular, verrucous lesions with raspberry-like growths	Face, scalp, mouth, nose, axilla, anogenital regions

Our patient had neurosyphilis presenting with meningeal signs. Although classically thought to occur late in the disease process, neurosyphilis can in fact occur at any stage [11]. There is currently no gold standard for the diagnosis of neurosyphilis, but in practice, it typically consists of a combination of clinical findings, CSF abnormalities, and serologic testing [11]. Nonspecific clinical symptoms of neurosyphilis include headaches, paresthesia, focal neurological deficits, or ataxic gait [8]. A classic symptom of neurosyphilis is Argyll-Robertson pupils, which are small irregular pupils that accommodate but do not react to light [8]. Argyll-Robertson pupils are thought to occur due to damage to the midbrain and manifest in up to 50% of patients with neurosyphilis, including our patient [8,12]. CSF abnormalities can include an elevated CSF protein, elevated WBC, positive VDRL test, or positive fluorescent treponemal antibody absorption (FTA-abs) test [11]. However, CSF VDRL and FTA-abs tests can be negative even in the presence of neurosyphilis [11,13].

Though our patient’s CSF VDRL test was negative, her clinical symptoms, positive syphilis serology, and CSF findings of elevated CSF protein and WBC confirmed the diagnosis of neurosyphilis. Treatment consists of intravenous or intramuscular penicillin G for 10-14 days, which our patient received. Our patient’s presentation shows that syphilis is a dynamic disease that is not always easy to define as the wide array of systemic manifestations varies.

Ocular syphilis is a specific form of neurosyphilis that occurs when *Treponema pallidum* invades the conjunctiva, cornea, sclera, choroid, retina, optic nerve, and blood vessels of the eye [13]. The most common presentation is syphilitic uveitis, as was the case in our patient [13]. The diagnosis of ocular syphilis is based on the combination of clinical findings on ophthalmologic testing with serologic tests positive for syphilis [13]. The treatment of ocular syphilis is similar to that of other types of neurosyphilis (intravenous or intramuscular penicillin G for 10-14 days), but corticosteroid eye drops can also be added to prevent irritation and reduce inflammation, as in our patient [13].

It is important to remember that when syphilis is a differential diagnosis, it can present in an atypical fashion, such as in our patient. Our patient had previously been seen by another physician, who attributed her symptoms to a CMV infection. Treatment with antiviral medications did not improve her symptoms. When our patient presented to the hospital, she was thought to have a rickettsial or parasitic infection, but a careful reconsideration of the different ways in which syphilis can present later made it our top differential diagnosis. The patient’s dermatological findings, neurological symptoms, and serologic confirmation helped us to establish the diagnosis of syphilis and treat her appropriately. It is likely that the scientific community has not discovered all the different ways in which syphilis can be present, so further clinical and molecular studies need to be done to enhance our understanding of syphilis and improve patient outcomes.

## Conclusions

Herein, we have discussed a rare presentation of serology-confirmed syphilis, notable for an extensive rash curiously sparing the palms and soles. This may represent a diffuse variant of follicular syphilis, where lesions occur around hair follicles, which are not typically present on the palms and soles. However, it is difficult to elucidate the definite reason for this unusual presentation. This case demonstrates the varied ways in which syphilis can manifest and the importance of maintaining it as a differential diagnosis when encountering rashes of unknown etiology. Ultimately, this case illuminates the need for further research into the mechanisms of the varied presentations of syphilis in order to inform the diagnostic process and ultimately improve timely management.
